# FBXO9 promotes anti-tumor immunity via degradation of PD-L1 in pancreatic cancer

**DOI:** 10.3389/fimmu.2025.1726825

**Published:** 2026-01-21

**Authors:** Tong Cao, Rui Zheng, Yingying Wang, Zixuan Yu, Hua Huang, Yufo Chen, Xueshan Pan, Yu Gao, Wenqing Song, Jun Xia, Hui Xu, Jia Ma

**Affiliations:** 1 Department of Clinical Laboratory, the First Affiliated Hospital of Bengbu Medical University, Bengbu, Anhui, China; 2 Bengbu Medical University Key Laboratory of Cancer Research and Clinical Laboratory Diagnosis, Bengbu Medical University, Bengbu, Anhui, China; 3 School of Clinical Medicine, Bengbu Medical University, Bengbu, Anhui, China; 4 School of Laboratory Medicine, Bengbu Medical University, Bengbu, Anhui, China; 5 Department of Medical Oncology, The First Affiliated Hospital of Bengbu Medical University, Bengbu, Anhui, China; 6 School of Life Science, Bengbu Medical University, Laboratory Animal Center, Bengbu, Anhui, China; 7 Department of Pathology, Bengbu Medical University, Bengbu, Anhui, China

**Keywords:** degradation, FBXO9, immunotherapy, pancreatic cancer, PD-1, PD-L1

## Abstract

Immune checkpoint blockade therapy, particularly those targeting programmed death 1/programmed cell death ligand 1 (PD-1/PD-L1), has been extensively employed to treat various human cancers, significantly improving clinical outcomes. Increasing evidence reveals that the therapeutic efficacy of PD-1/PD-L1 inhibitors depends on the abundance of PD-L1 on cancer cells and tumor-associated stromal cells. Here, we demonstrated that F-box protein 9 (FBXO9) is a novel regulator of PD-L1. We found that increased expression of FBXO9 suppresses tumor growth and promotes cytotoxic T cell activation *in vivo*. Mechanistically, FBXO9 directly binds to PD-L1 protein and enhances its degradation via ubiquitination, thereby impeding PD-L1 maturation and tumor immune evasion. Meanwhile, the expression of FBXO9 is decreased in pancreatic cancer tissues in comparison to normal tissues. Furthermore, FBXO9 expression correlates inversely with PD-L1 levels, with lower FBXO9 expression being associated with worse clinical outcome. These findings identify FBXO9 as a tumor suppressor via its facilitation of PD-L1 degradation, underscoring the potential of targeting FBXO9 in immunotherapeutic approaches for treating cancers, particularly in combination with anti-PD-L1 therapy.

## Introduction

Pancreatic cancer is highly lethal malignancy, ranking as the 7th leading cause of cancer-related deaths worldwide. Despite advancement in diagnostics, chemotherapy, radiotherapy and combination therapies, most patients with pancreatic cancer continue to exhibit poor survival rates (1–3). Thus, more effective treatments are urgently needed for this deadly disease. The advent of immunotherapy, particularly approaches targeting tumor-associated immune cells via the programmed death 1 (PD-1)/programmed cell death ligand 1 (PD-L1) axis, has emerged as promising frontier in cancer treatment (4). Immune checkpoint blockade therapy, a specific form of immunotherapy, overcomes mechanisms that impede immune recognition of cancer cells. Recent studies have demonstrated that PD-L1 is abnormally expressed in many tumor types, such as melanoma, acute myeloid leukemia, and colorectal cancer (5). PD-L1 inhibits CD8^+^ T cell activation and infiltration through engagement with PD-1, thereby allowing cancer cells to evade anti-tumor immunity (6). Previous studies have revealed that anti-PD-L1 immunotherapy can elicit robust anti-tumor immune responses and result in tumor regression (7).

The advent of PD-L1/PD-1 inhibitors has transformed the clinical management of cancers such as melanoma, lung cancer, and kidney cancer (8). However, the clinical response in other cancer types, including pancreatic cancer, remains suboptimal. For instance, in clinical trial, the objective response rate was only 3.1% for patients with metastatic pancreatic cancer receiving combination therapy, and 0% for those treated with Durvalumab monotherapy (9). Hence, thoroughly deciphering the regulatory mechanism of PD-L1 offers rationale for the prognostic prediction and therapeutic efficacy of immunotherapy in pancreatic cancer.

Compelling evidence suggests that the therapeutic efficacy of PD-L1 inhibitors can be markedly influenced by post-translational modifications (PTMs) (10, 11), especially ubiquitination. Ubiquitination drives a multitude of cellular processes, including the stabilization and interaction of proteins, which in turn governs cell proliferation, differentiation, and the dynamics of anti-tumor responses (12–14). Previous studies have indicated that ubiquitination and deubiquitination exert a pivotal function in the modulation of PD-L1 protein levels and its immunosuppressive functions (15, 16). For example, transmembrane and ubiquitin-like Domain containing 1 (TMUB1) enhanced PD-L1 stability by inhibiting its polyubiquitination at K281 in the endoplasmic reticulum, thereby promoting PD-L1 maturation and enhancing tumor immune evasion (17).

F-box proteins are substrate-recognition subunits of Skp1–Cullin–F-box (SCF) E3 ubiquitin ligase complexes that bind specific target proteins and promote their ubiquitination and proteasomal degradation (18, 19). F-box proteins have been identified to play a pivotal role in tumorigenesis in numerous cancer types (20–22). FBXO9 has been reported to regulate the expression of several proteins, such as Neurogenin 2 (Neurog2) (23), protein arginine methyltransferase 4 (PRMT4) (24), and peroxisome proliferator activated receptor gamma (PPARγ) (25). FBXO9 controls pluripotency via promotion of ubiquitylation and degradation of DPPA5 (26). FBXO9 degrades F-box and WD repeat domain containing 7 (FBXW7) and mediated zinc finger protein 143 (ZNF143)-induced cancer promotion as well as enhances drug resistance in hepatocellular carcinoma (27). Emerging evidence has pinpointed that FBXO9 is key factor in cell proliferation, metastasis and malignant progress in acute myeloid leukemia (28, 29). However, whether FBXO9 is involved in regulating cancer immunotherapy remains unclear. In this study, we demonstrated that FBXO9 plays a role in regulating PD-L1 ubiquitination, thereby suppressing cancer cell evasion. Taken together, FBXO9 may represent a potential target for novel immunotherapeutic approaches.

## Methods

### Plasmids and sgRNAs

The cDNA encoding HA or Flag-tagged FBXO9 and PD-L1 were amplified and cloned into the pcDNA3.1 vector for transfection. Additionally, they were cloned into pCDH lentiviral constructs for establishing stable cell lines. The FBXO9 △F-box was obtained from Youbio Biological Technology (Hunan, China). FBXO9 sgRNAs were purchased from GenePharma Company (Shanghai, China).

### Cell culture, transfection and generating stable cell lines

All cell lines were acquired from the Type Culture Collection of the Chinese Academy of Sciences (Shanghai, China), and maintained in DMEM containing 10% FBS. Lentiviruses overexpressing FBXO9 were used to generated stable cell lines of Panc-1 and Panc-02. The efficiency of transfection was determined by immunoblotting and RT-qPCR, which analyzed protein and mRNA expression, respectively.

### Animal models

All animal experiments were approved by the Animal Care and Use Committee of Bengbu Medical University. Nude mice or C57BL/6 mice were subcutaneously injected with 5×10^6^ Panc-1 or Panc-02 cells overexpressing FBXO9. Tumor dimensions were recorded at seven-day intervals (length × width), and tumor volume was calculated using the formula: *V=0.5 × Length × Width²*. After the experiments, tumors were harvested from the nude mice and subjected to immunoblot analysis. Tumors from the C57BL/6 mice were analyzed using immunoblotting and flow cytometry (FACS).

### Patients and tissues

Human pancreatic cancer tissues and adjacent normal tissues were provided by the Department of Pathology at the First Affiliated Hospital of Bengbu Medical University (Anhui, China) with the approval from the Medical Ethics Committee. FBXO9 and PD-L1 expression levels were analyzed by immunohistochemical (IHC) staining.

### Immunohistochemical staining

Briefly, tissues sections were deparaffinized and rehydrated, following by boiling in sodium citrate antigen retrieval solution for 15 minutes. After blocking, tissue slides were incubated with the indicated antibodies. Subsequently, DAB was added to slides for visualizing immunoreactivity. Representative images of each tissue were captured and analyzed. IHC staining was evaluated using a semi-quantitative scoring system: score = percentage of positively stained tumor cells (0: 0%; 1: 1–25%; 2: 26–50%; 3: 51–75%; 4: 76–100%) × staining intensity (0: negative; 1: weak; 2: moderate; 3: strong). For statistical analysis, a final score ≥4 was defined as high FBXO9 expression as described previously (30).

### Immunoblot and immunoprecipitation

For IB, cells were harvested and lysed in RIPA buffer containing 1% PMSF. Lysates were purified by centrifugation at 12,000 rpm for 15 minutes, after which the supernatants were collected. Proteins were separated by SDS-PAGE and transferred into PVDF membranes. After blocking in TBST, the PVDF membranes were immunoblotted with the indicated antibodies. Protein expression was visualized using the ECL buffer (31). For IP, total proteins were lysed in NP40 buffer with protease inhibitors. After centrifugation, 1–2 mg of whole cell lysate (WCL) was incubated with bead-conjugated anti-HA or anti-Flag antibodies overnight at 4°C. Subsequent immunoblotting was performed as described above. Protein levels were quantified by the ImageJ software (32).

### Protein half-life analysis

FBXO9-overexpressing cells were incubated with 400 μg/ml cycloheximide (CHX) for the indicated time points, after which the cells were collected for immunoblot analysis. The abundance of PD-L1 was normalized to β-actin levels.

### Ubiquitination assay

Cells were co-transfected with His-tagged ubiquitin (His-Ub) and the desired plasmids, then incubated with 10 μM MG132 overnight. Afterward, cells were lysed in NP40 lysis buffer, and the lysates were incubated with Ni–NTA beads. Finally, the immunoblotting was employed to detect ubiquitinated PD-L1 using anti-HA antibody.

### CCK8 assay

Cells were grown in 96-wells plate and incubated for 24 h, 48 h and 72 h. Subsequently, 10μl of CCK8 solution was added into each well, and cell viability was measured at OD450 after incubation (33).

### Wound healing assay

Briefly, transfected cells were seeded into 6-well plates and incubated for 24 h. A vertical scratch was made using a pipette tip, and images were captured at 0 and 20 h after the scratch to assess wound closure.

### Transwell migration and invasion assay

Cell capacities of migration and invasion were evaluated using Transwell assay. Transfected cells, cultured in 5% FBS, were seeded into Transwell chambers containing 10% FBS in a 24-well plate. For the invasion assay, the Transwell chambers were precoated with Matrigel before transfected cells were seeded. After incubation for 24 h, the cells that migrated or invaded through the membrane were stained, counted, and imaged under a microscope.

### RT-qPCR analysis

Total RNA was extracted from cells using the FastPure Complex Tissue/Cell Total RNA Isolation Kit (Vazyme, Nanjing, China). The HiScript III RT SuperMix for qPCR kit (Vazyme, Nanjing, China) was used to synthesize cDNA. RT-qPCR was performed using Bio-Rad CFX96 (Bio-Rad, USA) with ChamQ Universal SYBR qPCR Master Mix kit (Vazyme, Nanjing, China). Relative quantities of mRNA levels were normalized to GAPDH and calculated by the 2^-ΔΔCT^ method. The sequences of the primers employed for RT-qPCR were showed as follows, GAPDH: forward 5′-CAG CCT CAA GAT CAG CA-3′, reverse 5′-TGT GGT CAT GAG TCC TTC CA-3′; PD-L1: forward 5′-CAT TTG CTG AAC GCC CCA TA-3′, reverse 5′-TGT CCA GAT GAC TTC GGC CT-3′; FBXO9: forward 5′-TTG ACA ACC CCT GAA GAG CC-3′, reverse 5′-GTG ACA AGC GAT AGT GAC CC-3′.

### Flow cytometry

To ascertain the abundance of membrane PD-L1, cells were collected and incubated with APC- or PE-conjugated PD-L1 antibody, along with an appropriate isotype control, for 30 minutes. Subsequently, the samples were analyzed using flow cytometry, and data analysis was performed with FlowJo software. Median fluorescence intensity (MFI) was employed to measure the membrane abundance of PD-L1. For apoptosis detection, cells were stained with PE-annexin V and 7-AAD, and the apoptotic cell population was quantified by flow cytometry. Tumors were dissociated into single-cell suspensions, stained with Live/Dead dye and antibodies to CD45, CD3, CD4, and CD8, and analyzed by flow cytometry. Debris and doublets were excluded, then live CD45^+^ cells were gated, followed by CD3^+^ T cells, and finally CD8^+^ T-cell subsets for analysis.

### Statistical analysis

Statistical analyses were performed by GraphPad Prism 8 software (GraphPad Software, Inc.). Two-tailed unpaired Student’s *t*-test and paired *t-*test were used to compare differences between two groups. Kaplan-Meier analyses with the log-rank Mantel-Cox test was conducted for survival analysis. *P*-values < 0.05 were considered statistically significant.

## Results

### Reduced FBXO9 is closely correlated with poor prognosis in pancreatic cancer

**Figure 1:**
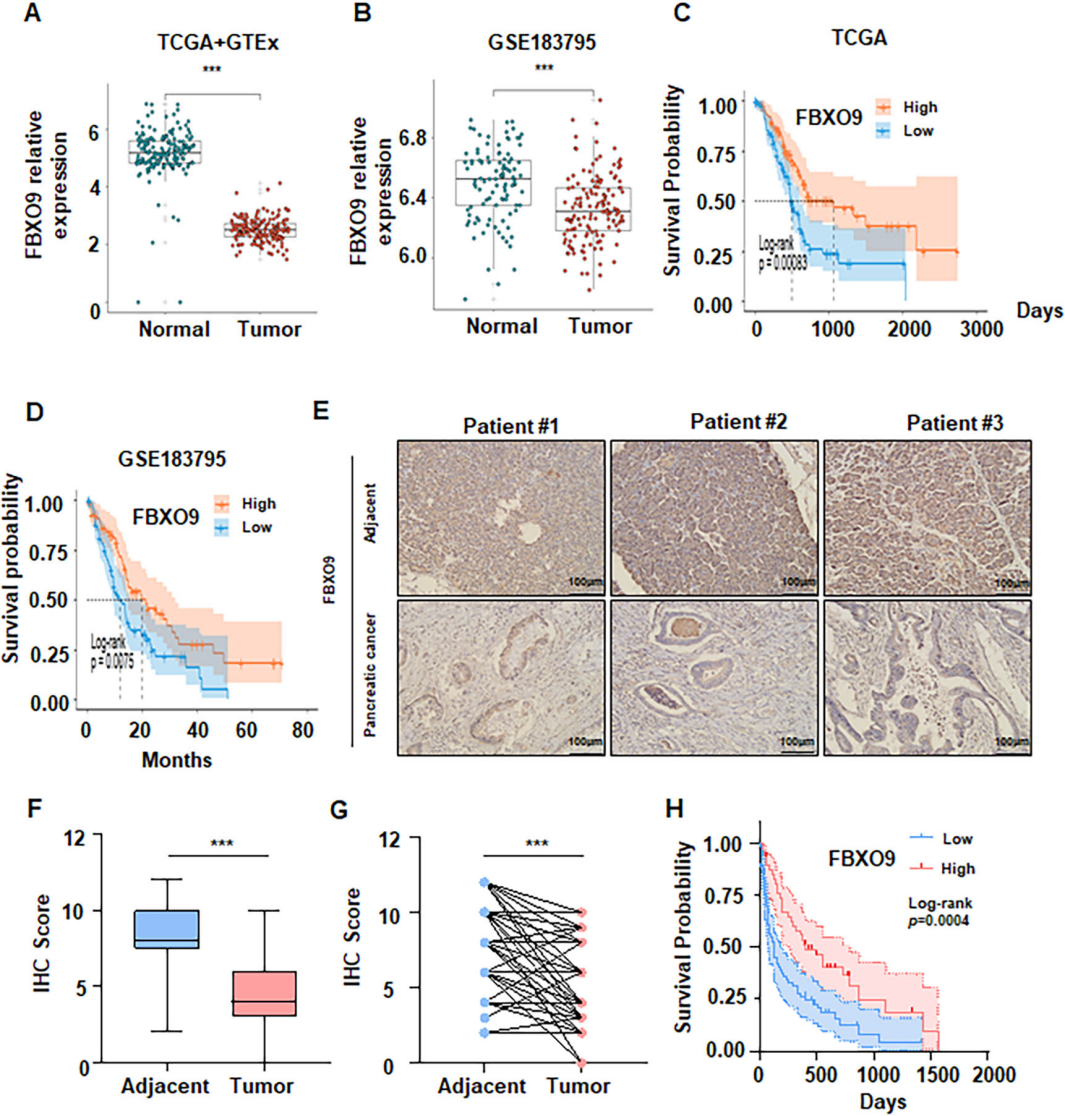
Reduced FBXO9 expression is associated with poor clinical outcomes in pancreatic cancer patients. **(A, B)** FBXO9 expression was significantly decreased in pancreatic cancer tissues compared to normal tissues based on the TCGA and GEO databases (GEO ID: GSE183795). ***p < 0.001 compared to normal tissues. **(C, D)** Kaplan-Meier survival analysis of pancreatic cancer patients from TCGA and GEO databases. **(E)** Representative IHC staining showed a significant decrease in FBXO9 protein expression in pancreatic cancer tissues compared to adjacent normal tissues. scale bar: 100 μm. **(F)** Comparison of FBXO9 expression between pancreatic cancer tissues (n=112) and adjacent tissues (n=82). **(G)** FBXO9 expression in paired tissue samples from pancreatic cancer patients (n=82). ***p < 0.001 compared to adjacent tissues. **(H)** Kaplan-Meier curve for overall survival of pancreatic cancer patients based on FBXO9 expression scores, as calculated by IHC staining. Patients were divided into a high FBXO9 group (n=46) or a low FBXO9 group (n=66).

To explore the role of FBXO9 in pancreatic cancer, we initially utilized The Cancer Genome Atlas (TCGA) database and GSE183795 cohort for bioinformation analysis. Our results demonstrated that FBXO9 expression is markedly decreased in pancreatic cancer tissues in comparison to normal tissues. (**Figures 1A, B**). We further analyzed the relationship between FBXO9 and the clinical prognosis of pancreatic cancer patients based on data from the TCGA database, the GSE183795 cohort, and IHC analysis. The results revealed that lower expression of FBXO9 was correlated with inferior overall survival (**Figures 1C, D**). Additionally, IHC staining was confirmed a reduction in the expression of FBXO9 in tumor tissues compared with adjacent normal tissues (**Figures 1E, F**). In paired tissue samples, FBXO9 was also observed to be downregulated in tumor tissues relative to adjacent tissues (**Figure 1G**). Furthermore, overall survival analysis demonstrated that individuals with low levels of FBXO9 exhibited markedly worse overall survival (**Figure 1H**). Additionally, a consistent association was identified between lower FBXO9 expression and TNM stage and other clinical parameters in the pancreatic cancer patient cohort (**Table 1**). These findings suggest that reduced FBXO9 expression is associated with an unfavorable prognosis in patients with pancreatic cancer.

**Table 1:** Clinicopathological relevance of FBXO9 in pancreatic cancer.

Characteristics	FBXO9 low	FBXO9 high	X^2^	P value
Age, years				
≥60	48 (72.73%)	25 (54.35%)		
<60	18 (27.27%)	21 (45.65%)	4.035	0.045
Gender				
Female	30 (45.45%)	20 (43.48%)		
Male	36 (54.55%)	26 (56.52%)	0.043	0.836
Serum CA19-9				
≥37 U/ml	50 (75.76%)	33 (71.74%)		
<37 U/ml	16 (24.24%)	13 (28.26%)	0.228	0.633
Serum CEA				
≥5 U/ml	24 (36.36%)	17 (36.96%)		
<5 U/ml	42 (63.67%)	29 (63.04%)	0.004	0.949
TNM stage				
I	20 (30.30%)	21 (45.65%)		
II	14 (21.21%)	15 (32.61%)		
III	9 (13.64%)	3 (6.52%)		
IV	23 (34.85%)	7 (15.22%)	8.273	0.004
Distant metastasis				
M0	43 (65.15%)	39 (84.78%)		
M1	23 (34.85%)	7 (15.22%)	5.327	0.021
Nerve invasion				
Yes	22 (33.33%)	15 (32.61%)		
No	44 (66.67%)	31 (67.39%)	0.006	0.936

### FBXO9 inhibits cell growth and tumorigenesis

**Figure 2:**
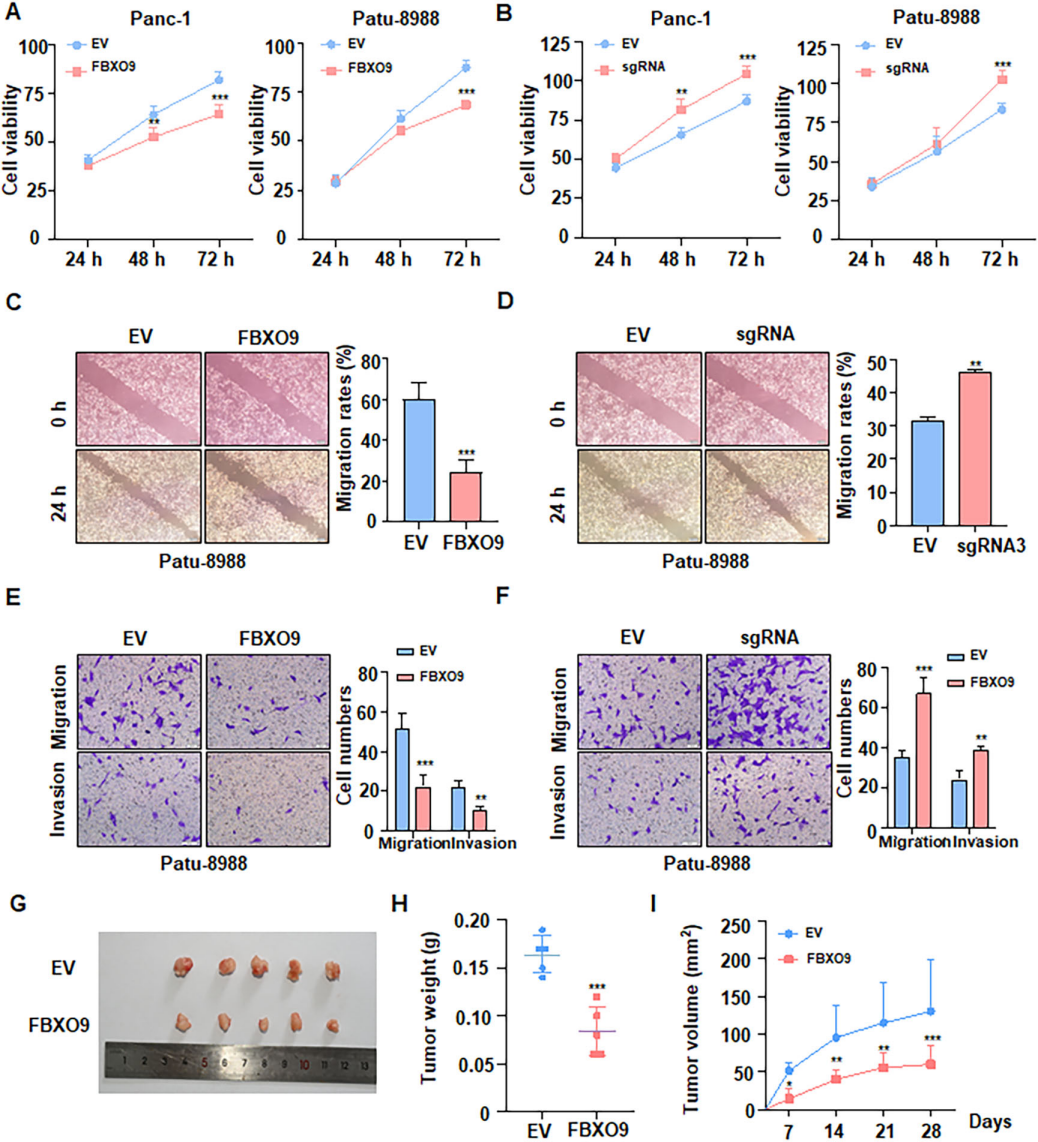
FBXO9 inhibits cell growth and tumorigenesis. **(A, B)** CCK8 assays showing cell growth of Panc-1 and Patu-8988 cells transfected with either FBXO9 sgRNA **(A)** or FBXO9 overexpression plasmid **(B)**. ***p < 0.001 compared to the EV control group. **(C, D)** Wounding healing assays to analyze the migration of Patu-8988 cells transfected with FBXO9 sgRNA **(C)** or FBXO9 overexpression plasmid **(D)**. **p < 0.01, ***p < 0.001 compared to the EV control group. **(E, F)** Transwell assays measuring migration and invasion of PATU-8988 cells transfected with FBXO9 sgRNA **(E)** or FBXO9 overexpression plasmid **(F)**. **p < 0.01, ***p < 0.001 compared to the EV control group. **(G)** Representative images of tumors mass dissected from FBXO9-overexpressing xenografts mouse models. Stable FBXO9-overexpressing Panc-1 cells and control cells were injected subcutaneously into the BALB/c nude mice to establish xenografts models. **(H, I)** Statistic results of tumor weight **(H)** and volume **(I)** from the xenograft tumors from xenografts shown in **(G)** **p < 0.01, ***p < 0.001 compared to the EV control group.

To clarify the biological role of FBXO9 in pancreatic cancer, knockdown and overexpression experiments were conducted in Panc-1 and Patu-8988 cell lines, respectively (**Supplementary Figures 1A, B**). Then, using CCK-8 assays, we detected cell viability and revealed that FBXO9 overexpression inhibited cell viability, whereas FBXO9 silencing enhanced cell viability (**Figures 2A, B**). To assess cell migration and invasion ability, wound healing and Transwell assays were performed in pancreatic cancer cells after transfection. As expected, the capacity of migration and invasion was significantly attenuated by the overexpression of FBXO9 (**Figures 2C-F**, **Supplementary Figures 2A-D**). To further investigate the anti-tumor potential of FBXO9 *in vivo*, subcutaneous implantation of FBXO9-overexpressing Panc-1 cells was performed in nude mice. We found that FBXO9-overexpression significantly delayed tumor growth and reduced tumor weight (**Figures 2G-I**). Taken together, these data suggest that FBXO9 suppresses pancreatic tumorigenesis.

### FBXO9 activates an anti-tumor immune response

**Figure 3:**
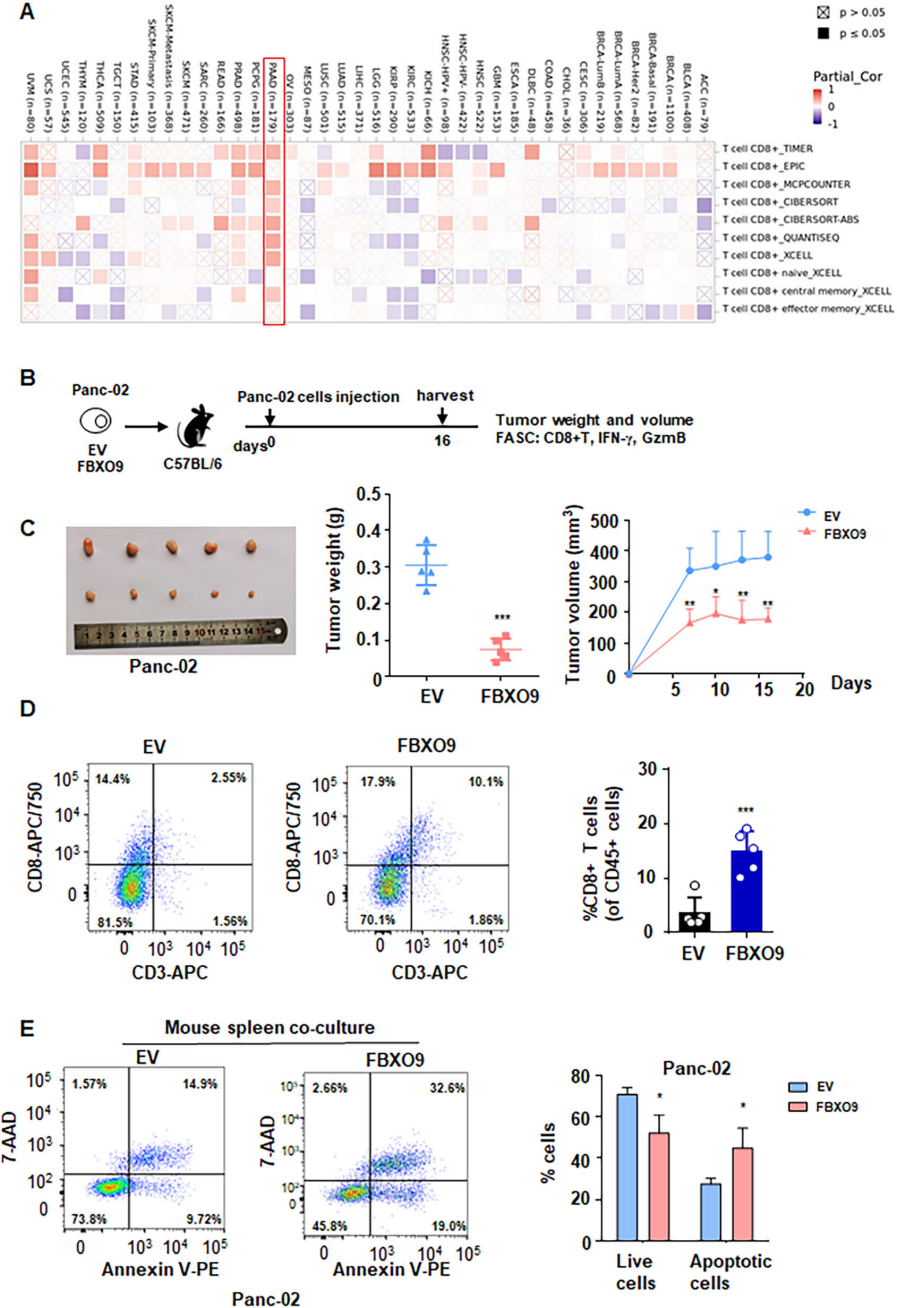
FBXO9 activates an anti-tumor immune response. **(A)** Bioinformatics analysis of the correlation between FBXO9 and immune effector cells using the TIMER database. **(B)** Schematic showing the experimental protocol in which EV and FBXO9-overexpressing Panc-02 cells were separately injected subcutaneously into immunocompetent C57BL/6 mice. **(C)** Representative images of tumors, weights and growth curves from C57BL/6 mice. *p < 0.05, **p < 0.01, ***p < 0.001 compared to EV control group. **(D)** Flow cytometry analysis of the infiltration of CD8^+^ T cells derived from tumors. **p < 0.01 compared to the EV control group. **(E)** Flow cytometry analysis of apoptosis in Panc-02 cells cocultured with splenic cells from healthy C57BL/6 mice, *p < 0.05 compared to the EV control group.

To evaluate whether FBXO9 suppresses tumorigenesis via immune responses, we analyzed the correlation between FBXO9 and immune effector cells by using the TIMER database (http://timer.cistrome.org/). The analysis revealed a close relationship between FBXO9 expression and CD8**^+^** T cells in pancreatic cancer (**Figure 3A**, **Supplementary Figures 3A-C**), suggesting that FBXO9 may play an immunoregulatory role in the tumor microenvironmental of pancreatic cancer. To further explore the effects of FBXO9 in anti-tumor immune response, we upregulated FBXO9 in Panc-02 mouse pancreatic cancer cells and then transplanted them into immunocompetent C57BL/6 mice (**Figure 3B**). In comparison with the EV control group, tumors derived from FBXO9-overexpressing cells showed significantly reduced growth in C57BL/6 mice (**Figure 3C**). Moreover, tumor weight was lower in the FBXO9 overexpression group in comparison to the EV control group (**Figure 3C**). Flow cytometry further revealed a notable elevation in CD8+ T cell infiltration in FBXO9-overexpressing tumors (**Figure 3D**). Additionally, Panc-02 cells with FBXO9 overexpression or EV control cells were cocultured for 3 days with splenic cells from healthy C57BL/6 mice. Apoptosis was significantly increased in FBXO9-overexpressing Panc-02 cells compared with EV control group (**Figure 3E**). Collectively, these results demonstrated that FBXO9 promoted anti-tumor immunity.

### FBXO9 downregulates PD-L1 protein in pancreatic cancer

**Figure 4:**
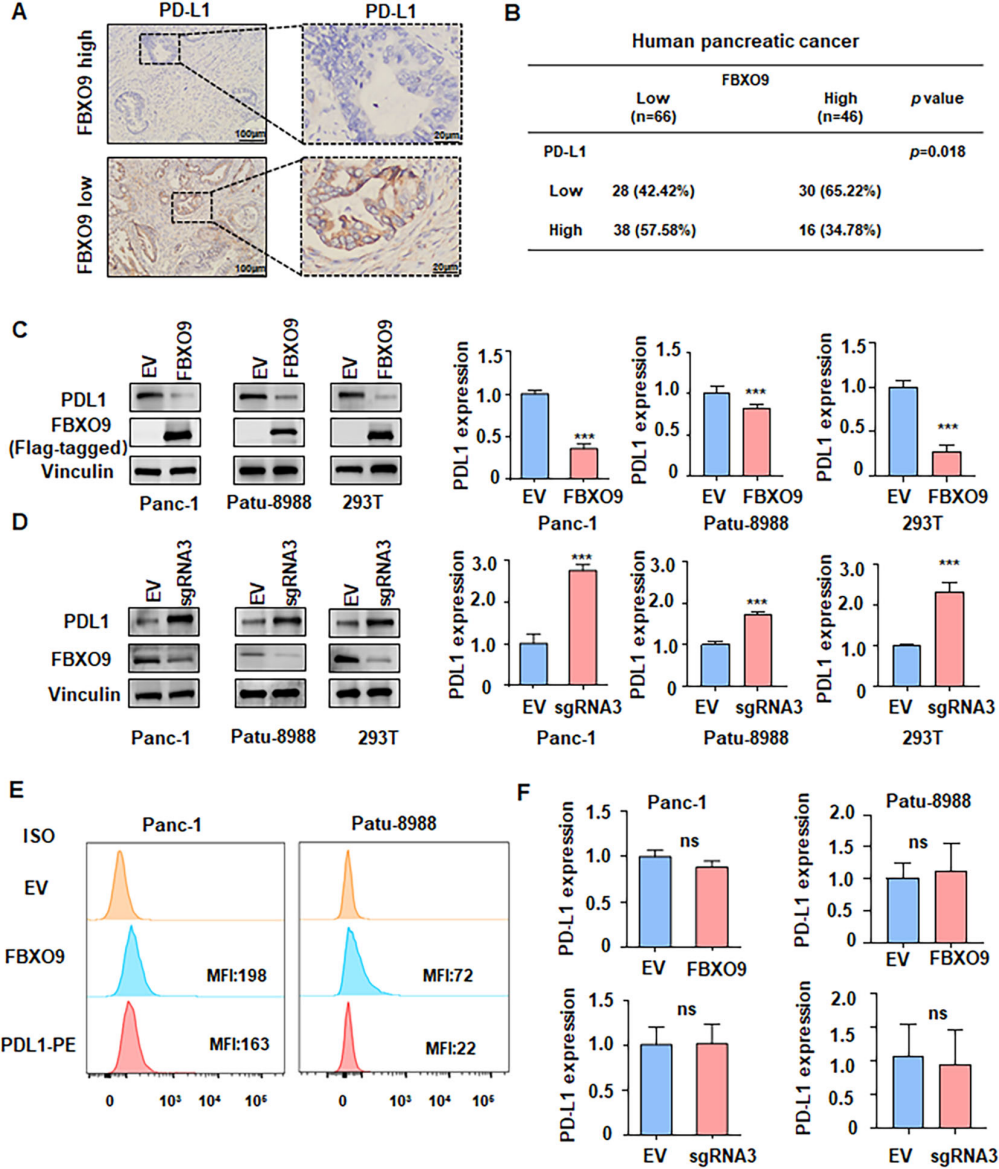
FBXO9 downregulates PD-L1 protein level. **(A)** Representative IHC staining of pancreatic cancer tumors showing PD-L1 expression. **(B)** Correlation between FBXO9 and PD-L1 protein levels in pancreatic cancer tissues from our cohort. **(C, D)** Immunoblot analysis of PD-L1 protein levels in Patu-8988, Panc-1 and 293T cells after transfection with FBXO9 overexpression plasmid or FBXO9 sgRNA. Quantitative results are shown on the right panel. ***p < 0.001 compared to the EV control group. **(E)** Flow cytometry analysis of PD-L1 abundance on the surface of pancreatic cancer cells after transfection with FBXO9 overexpression plasmid. **(F)** RT-qPCR analysis of PD-L1 mRNA levels in Patu-8988, and Panc-1 cells after transfection with FBXO9 overexpression plasmid or FBXO9 sgRNA. ns, not significant.

To evaluate the clinical relevance of FBXO9 and PD-L1, we examined their protein expression levels in tumor tissues from patients with pancreatic cancer using IHC. The analysis revealed a negative correlation between FBXO9 and PD-L1 expression. (**Figures 4A, B**). Next, we investigated whether FBXO9 directly affects PD-L1 protein level. Expectedly, FBXO9 overexpression led to a remarkable decrease in PD-L1 protein levels in Panc-1, Patu-8988, and 239T cells (**Figure 4C**). Conversely, FBXO9 knockdown markedly increased PD-L1 protein levels (**Figure 4D**). Flow cytometry further confirmed a noticeable reduction in cell membrane PD-L1 levels in the presence of FBXO9 overexpression (**Figure 4E**). In addition, it is noteworthy that PD-L1 mRNA level were not altered with the changes in FBXO9 expression revealed by RT-qPCR analysis (**Figure 4F**), suggesting that FBXO9 may regulate PD-L1 largely at post-transcriptional level, likely through the ubiquitin-proteasome system.

### FBXO9 facilitates PD-L1 degradation via ubiquitination

**Figure 5:**
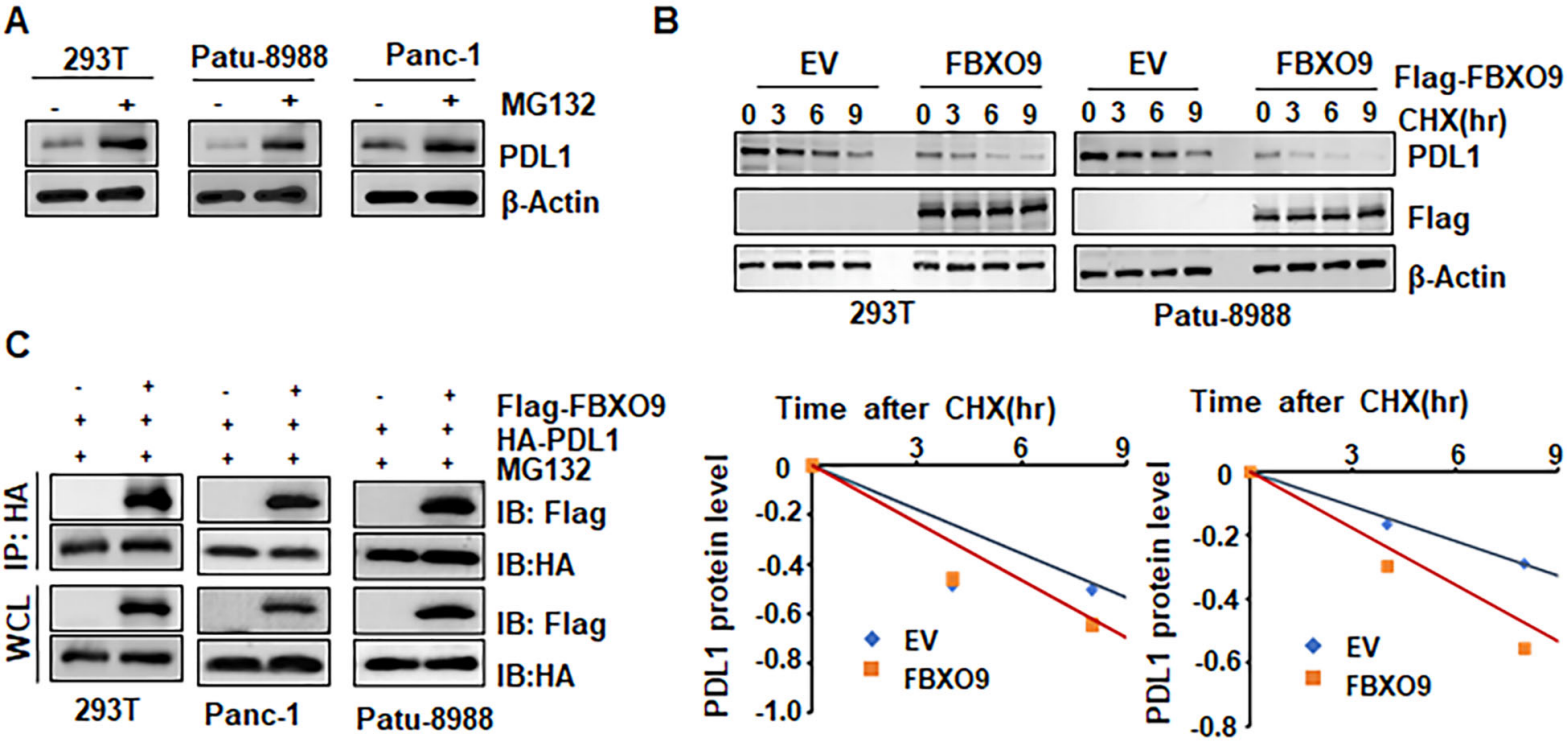
FBXO9 facilitates PD-L1 degradation. **(A)** Immunoblot (IB) analysis of whole cell lysates (WCLs) derived from 293T, Patu-8988 and Panc-1 cells treated with MG132. **(B)** IB analysis of WCLs derived from 293T, Patu-8988 cells transfected with FBXO9 constructs (Top panel). Cells were treated with 100 mg/ml cycloheximide (CHX) for the indicated time points. PD-L1 protein abundance was quantified and plotted (Bottom panel). **(C)** IB analysis of WCLs and anti-HA immunoprecipitations (IPs) from 293T, Panc-1, and Patu-8988 cells co-transfected with the indicated plasmids. Cells were treated with 10 μM MG132 for 6 h before harvesting.

**Figure 6:**
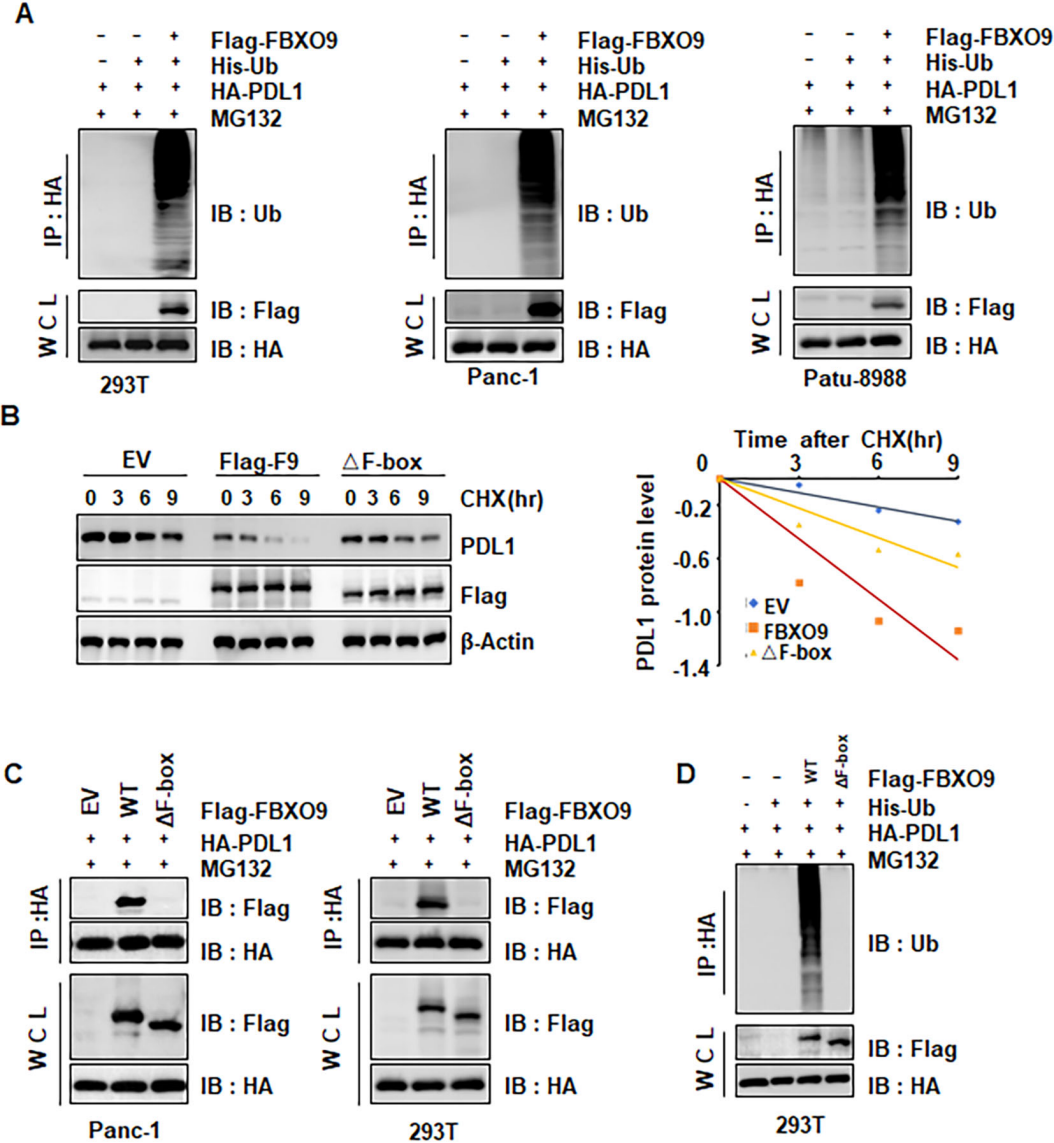
FBXO9 induces PD-L1 degradation via ubiquitination. **(A)** IB analysis of products of ubiquitination and WCLs derived from lysates of 293T, Panc-1, and Patu-8988 cells transfected with the indicated constructs. **(B)** IB analysis of WCLs derived from Panc-1 cells transfected with indicated constructs. Cells were treated with 100 μg/ml CHX for the indicated time points. PD-L1 protein abundance was quantified and plotted. **(C)** IB analysis of IPs and WCLs derived from Panc-1 and 293T cells transfected with the indicated plasmids. Cells were treated with 10 μM MG132 for 6 h before harvesting. **(D)** IB analysis of products of ubiquitination and WCLs derived from 293T cells transfected with the indicated constructs.

Since the ubiquitin E3 ligase FBXO9 promotes substrate proteins ubiquitination and subsequent degradation, we investigated whether FBXO9 promotes PD-L1 ubiquitination and degradation. We found that the proteasome inhibitor MG132 enhanced PD-L1 protein accumulation, indicating that the proteasome pathway is responsible for FBXO9-mediated PD-L1 degradation (**Figure 5A**). Next, we further investigate the regulatory role of FBXO9 in PD-L1 protein stability. We found that ectopic overexpression of FBXO9 dramatically shorten the half-life of PD-L1 protein (**Figure 5B**), suggesting that FBXO9 degrades PD-L1 protein in pancreatic cancer cells. Consistently, the interaction between FBXO9 and PD-L1 was observed in multiple cell lines (**Figure 5C**). Notably, *in vivo* ubiquitination assays demonstrated that FBXO9 overexpression dramatically elevated PD-L1 poly-ubiquitination (**Figure 6A**). These findings reveal that FBXO9 directly interacts with PD-L1, thereby promoting its ubiquitination and degradation.

The F-box domain is responsible for directing the ubiquitination of substrates, which is essential for the regulation of numerous cellular functions (34). To determine whether the F-box domain mediates the interaction between FBXO9 and PD-L1, we generated an FBXO9 mutant deleting F-box (△F-box). As expected, the FBXO9 ΔF-box mutation increased the protein abundance of PD-L1 in comparison to wild-type FBXO9. In line with this, the protein half-life of endogenous PD-L1 was observed to be considerably prolonged in cells expressing the FBXO9 ΔF-box mutant (**Figure 6B**). Furthermore, the FBXO9 ΔF-box mutant disrupted the binding with PD-L1 (**Figure 6C**), and nearly abolished PD-L1 polyubiquitination (**Figure 6D**). These results reveled that the F-box domain of FBXO9 is necessary for PD-L1 binding and ubiquitination.

## Discussion

Immune system suppression and dysfunction are hallmarks of various cancers (35). However, new approaches aimed at reinvigorating anti-tumor immune responses are becoming increasingly prominent in cancer treatment. Immunotherapy, especially immune checkpoint blockade therapies such as anti-PD-1/PD-L1, has exhibited unprecedented clinical efficacy (36, 37). The effectiveness of these therapies is complicated and has been attributed to the abundance of PD-L1 in both tumor cells and T cells (38, 39). As such, elucidating the regulatory mechanism of PD-L1 is crucial for the advancement of novel cancer immunotherapy strategies. Recent studies have highlighted that different PTM mechanisms, including glycosylation, SUMOylation, phosphorylation and ubiquitination, play important roles in the regulation of PD-L1 protein stability, translocation and interactions (40–43). Aberrant alterations of PTMs can directly influence PD-L1-mediated immune resistance. Nevertheless, the mechanisms governing PD-L1 expression via the ubiquitin-proteasome system remain incompletely understood. Our study revealed that FBXO9 functions as a PD-L1 binding partner, directly ubiquitinating and mediating the degradation of PD-L1 in pancreatic cancer cells.

Several F-box proteins have been discovered to regulate the stability of PD-1 and PD-L1. For instance, FBXO38 governs anti-tumor immunity of T cells via targeting PD-1 for ubiquitination and degradation (44). However, one recent study showed that FBXO38 is dispensable for PD-1 regulation (45). FBW7 increases sensitivity of anti-PD-1 immunotherapy via promotion of ubiquitination and destruction of PD-1 in non-small cell lung cancer (46). FBXO22 has been reported to degrade PD-L1 and sensitize tumor cells to DNA damage (47). PD-L1 immunosuppression is regulated by N-glycosylation and glycogen synthase kinase 3 beta (GSK3β) and beta-transducin repeat–containing protein (β-TrCP)–mediated ubiquitination. Epidermal growth factor (EGF) inactivates GSK3β, stabilizing PD-L1, reducing antitumor T-cell responses and PD-1 blockade efficacy (48). Cyclin D–CDK4 and speckle-type POZ protein (SPOP) control PD-L1 degradation. CDK4/6 inhibition or SPOP loss stabilizes PD-L1, while combining CDK4/6 inhibitors with PD-1/PD-L1 blockade enhances antitumor efficacy (49). Vaccinia-related kinase 2 (VRK2) stabilizes MYC via Ser281/293 phosphorylation, blocking SCF–FBXO24-mediated ubiquitination, thereby upregulating PD-L1 and protumor programs in HCC (50). Several studies have demonstrated that FBXO9 functions as a tumor suppressor and could serve as prognostic biomarker (28, 51). Mechanisms supporting the tumor suppressive role of FBXO9 have been proposed. For instance, depletion of FBXO9 has been shown to reduce ATP6V1A ubiquitination, resulting in increased metastasis in lung cancer cells (52). In this study, we identified that FBXO9 can target PD-L1 for ubiquitination and degradation in pancreatic cancer.

Evidence reveals that lower FBXO9 levels are associated with poorer survival outcomes in patients with lung cancer (52). One study showed that FBXO9 and CK2 govern cellular response to growth factor depletion through degradation of Tel1/Tti1 and lead to survival in multiple myeloma (53). In ovarian cancer, FBXO9 expression is decreased and positively associated with DNA damage repair, indicating FBXO9 as a tumor suppressor in ovarian cancer (51). Consistently, our study illustrated that the expression of FBXO9 is decreased in pancreatic cancer and is strongly correlated with poor survival in patients with pancreatic cancer. *In vitro*, the ectopic overexpression of FBXO9 decreased cell viability, suppressed migration and invasion capability in pancreatic cancer cells. Besides, FBXO9 overexpression significantly retarded tumor growth *in vivo*.

The evidence presented here indicates that FBXO9 enhances the recruitment of CD8^+^ T cells, and that pancreatic cancer patients with high FBXO9 expression show significant downregulation of PD-L1. However, the precise mechanism by which FBXO9 modulates PD-L1 in the context of immunotherapy, especially at the post-translational level, remains poorly understood. In this study, we observed that FBXO9 negatively regulates the protein level of PD-L1, but not its mRNA level. FBXO9 could directly bind to PD-L1 through its F-box domain and facilitate PD-L1 ubiquitination and subsequent degradation. FBXO9 suppresses pancreatic cancer cell growth, migration, and invasion, suggesting that it may act on additional tumor-suppressive targets beyond PD-L1 that regulate intrinsic cancer cell behavior.

## Conclusion

In conclusion, our findings provide evidence that FBXO9 functions as a tumor suppressor by targeting PD-L1 for ubiquitination-dependent degradation. FBXO9 expression is inversely associated with PD-L1 levels. Moreover, lower expression of FBXO9 is correlated with worse clinical outcome. This identifies FBXO9 as a promising therapeutic target with the potential to improve the clinical efficacy of immunotherapy. The following limitations should be acknowledged. (1) It remains unclear whether additional F-box family members also regulate PD-L1 in pancreatic cancer. (2) Although the authors demonstrate that FBXO9 ubiquitinates PD-L1, the specific lysine residue(s) involved and the subcellular compartment in which this occurs (ER, Golgi, or plasma membrane) have not been elucidated. (3) Beyond CD8^+^ T cells, it is important to clarify whether Tregs, myeloid cells, or NK cells also contribute to FBXO9-mediated immunomodulation. (4) It would be valuable to investigate whether combining FBXO9 overexpression with anti–PD-1/PD-L1 therapy confers additive or synergistic therapeutic benefit in pancreatic cancer.

Future perspectives are outlined below. To date, whether FBXO9 enhances anti-tumor immunity remains incompletely understood. Since FBXO9 has multiple known substrates, it is necessary to investigate whether these additional substrates also influence anti-tumor immunity in pancreatic cancer. It will also be important to determine whether FBXO22, β-TrCP, and SPOP similarly target PD-L1 for degradation in pancreatic cancer. This study included 112 pancreatic cancer tissues for IHC analysis. However, it is critical to validate these findings in additional, independent clinical tissue cohorts. Moreover, future work should also assess FBXO9 overexpression combined with anti–PD-L1 treatment in syngeneic models to determine potential sensitization and therapeutic benefit in pancreatic cancer.

## Data Availability

The original contributions presented in the study are included in the article/**Supplementary Material**. Further inquiries can be directed to the corresponding authors.
