# Supplementary material for: FBXO9 promotes anti-tumor immunity via degradation of PD-L1 in pancreatic cancer
Source: Front Immunol. 2026 Jan 21;16:1726825. doi: 10.3389/fimmu.2025.1726825 (PMC12867838; doi:10.3389/fimmu.2025.1726825)
Supplement: Supplementary file 1 [file DataSheet1.pdf]

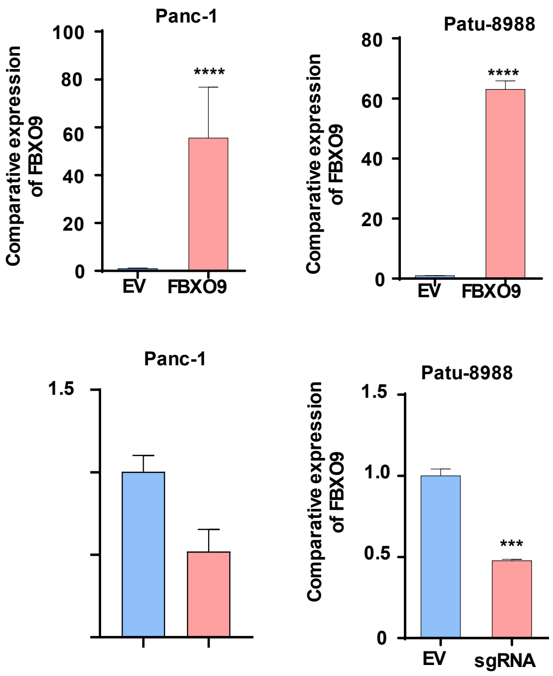

Supplementary figure 1: The transfection efficacy of FBXO9 overexpression plasmid and sgRNA. RT-qPCR was used to detected FBXO9 mRNA after transfected FBXO9 overexpression (A) and FBXO9 sgRNA (B) in Panc-1 and Patu-8988 cells.

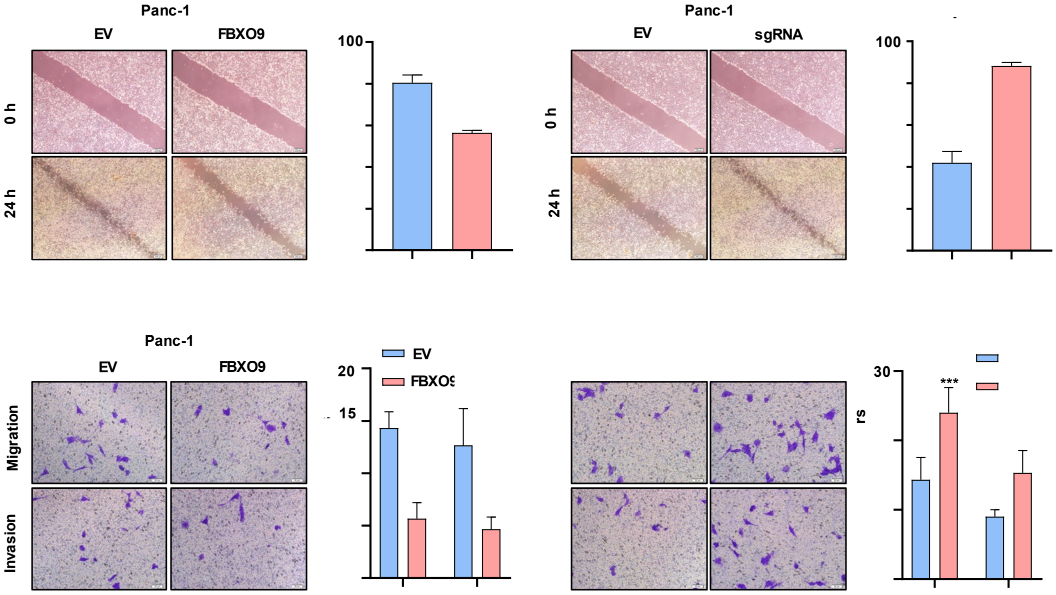

Supplementary figure 2: FBXO9 inhibited cell migration and invasion in PC cells. A-B: wound healing assays to analyze cell migration of PANC-1 cells after transfected with FBXO9 overexpression plasmid (A) or FBXO9 sgRNA (B). $**p < 0.01$, $***p < 0.001$ as compared to EV control group. C-D: Transwell assays to measure cell migration and invasion of PANC-1 cells after transfected with FBXO9 overexpression plasmid (C) or FBXO9 sgRNA (D). $**p < 0.01$, $***p < 0.001$ as compared to EV group.

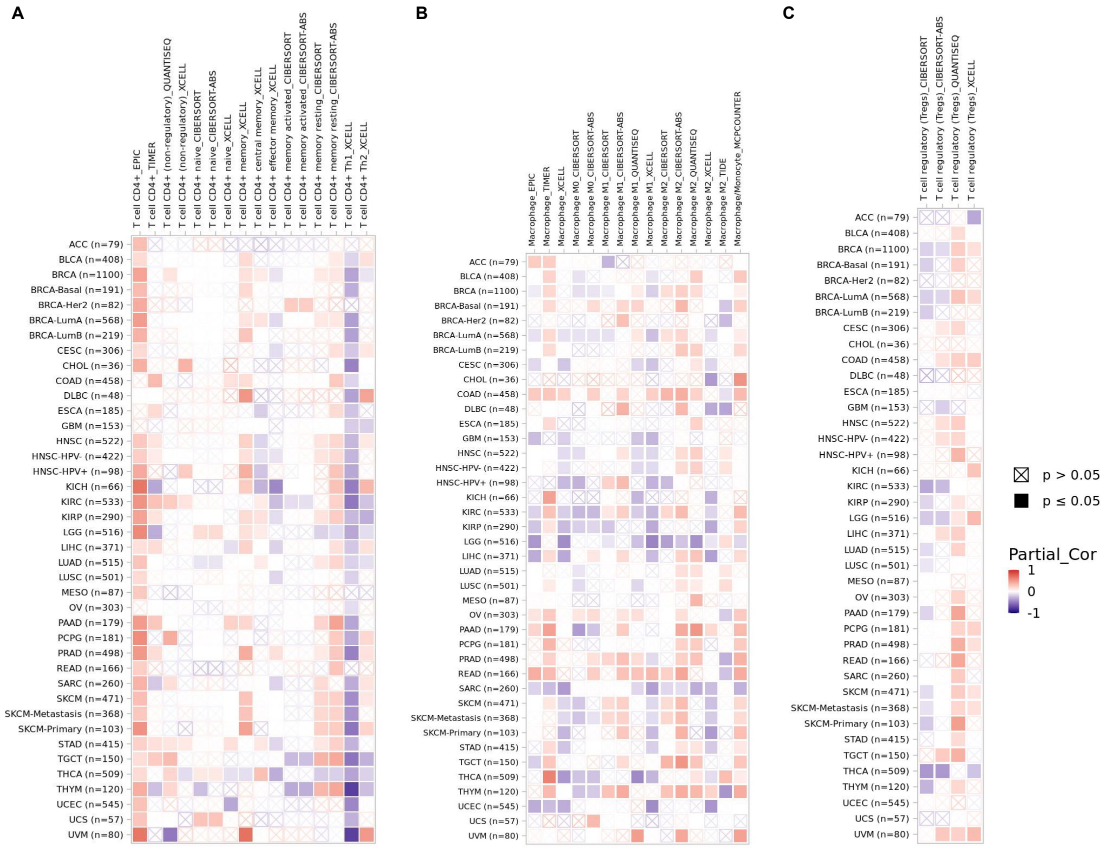

Supplementary figure 3: FBXO9 activates an anti-tumor immune response. A-C: Bioinformatics analysis of the correlation between FBXO9 and immune effector cells using TIMER.
